# Supplementary material for: Area postrema neurons mediate interleukin-6 function in cancer cachexia
Source: Nat Commun. 2024 Jun 1;15:4682. doi: 10.1038/s41467-024-48971-1 (PMC11144211; doi:10.1038/s41467-024-48971-1)
Supplement: Supplementary file 1 — Supplementary Information [file 41467_2024_48971_MOESM1_ESM.pdf]

Area postrema neurons mediate interleukin-6 function in cancer cachexia

Qingtao Sun[1,#], Daniëlle van de Lisdonk[1,2,#], Miriam Ferrer[1,#], Bruno Gegenhuber[1], Melody Wu[1], Youngkyu Park[1], David A Tuveson[1], Jessica Tollkuhn[1], Tobias Janowitz[1], Bo Li[1,3,4,5*]

1. Cold Spring Harbor Laboratory, Cold Spring Harbor, NY 11724, USA
2. Center for Neuroscience, University of Amsterdam, Amsterdam, the Netherlands
3. Westlake Laboratory of Life Sciences and Biomedicine, Hangzhou 310024, Zhejiang, China
4. School of Life Sciences, Westlake University, Hangzhou 310024, Zhejiang, China
5. Institute of Biology, Westlake Institute for Advanced Study, Hangzhou 310024, Zhejiang, China

# These authors contributed equally

*Correspondence: Bo Li (bli@cshl.edu; libo@westlake.edu.cn)

Supplementary figures, tables and legends

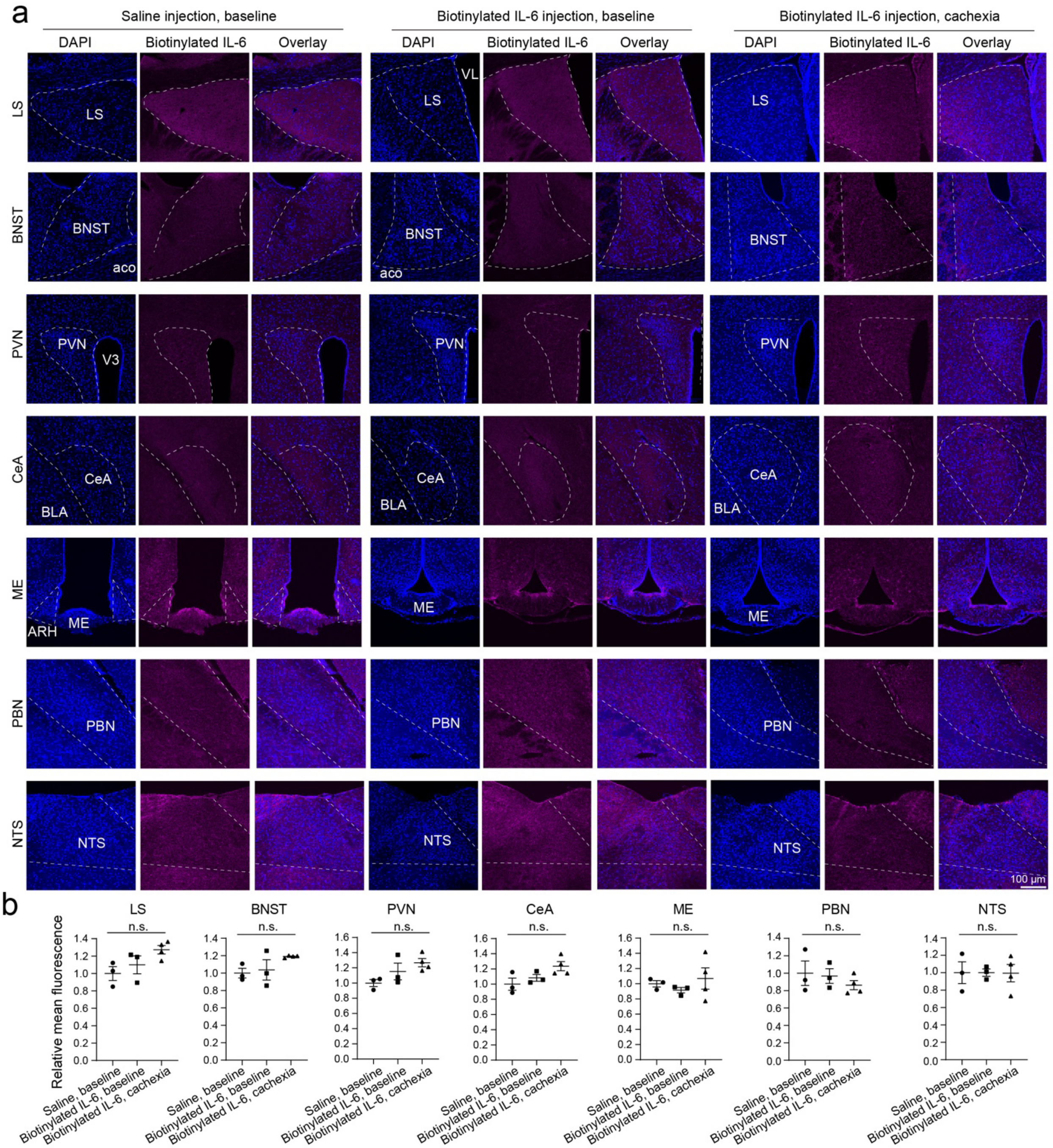

Supplementary Figure 1 | Circulating IL-6 does not reach brain areas other than the AP. a. Confocal immunohistochemical images showing the lack of exogenous IL-6 signals in different brain areas of mice received biotinylated IL-6 under baseline (middle) and cachexia (right) conditions compared with mice received saline under baseline condition (left) via retro-orbital injection. **b.** Quantification of the fluorescence signals from fluorescence-conjugated avidin in different brain areas, which recognizes the biotinylated exogenous IL-6 (n = 3, 3, and 4 mice in

saline baseline, IL-6 baseline, and IL-6 cachexia groups, respectively; LS, $F = 3.605$, $P = 0.084$; BNST, $F = 2.54$, $P = 0.15$; PVN, $F = 3.57$, $P = 0.086$; CeA, $F = 3.73$, $P = 0.08$; ME, $F = 0.55$, $P = 0.6$; PBN, $F = 0.66$, $P = 0.55$; NTS, $F = 0.0012$, $P = 0.98$; n.s., nonsignificant; one-way ANOVA). LS, lateral septum; BNST, bed nucleus of the stria terminalis; PVN, paraventricular nucleus of hypothalamus; CeA, central amygdala; ME, median eminence; PBN, parabrachial nucleus; NTS, nucleus tractus solitarii.

Data in b are presented as mean ± s.e.m. Source data are provided as a Source Data file.

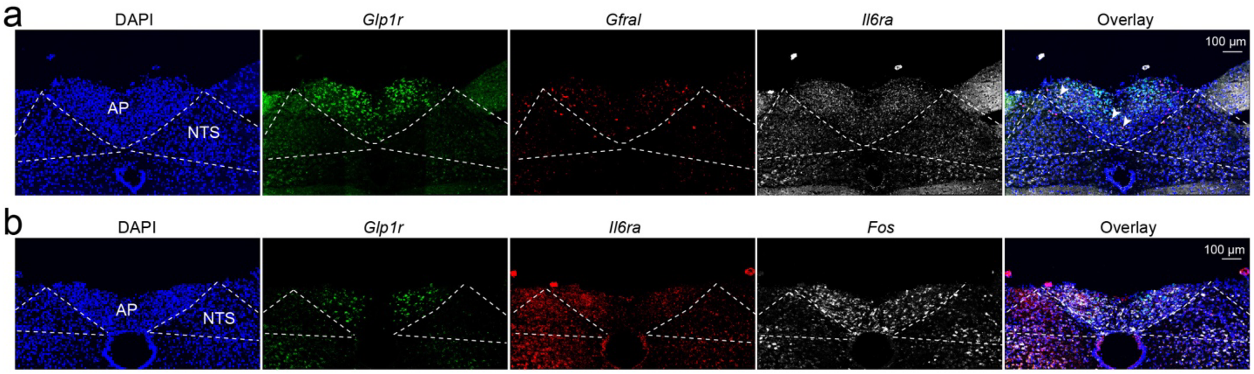

Supplementary Figure 2 | The expression of genetic markers in the AP and NTS. a. Confocal images showing the expression of different genes in AP and NTS cells, detected with single molecule fluorescent *in situ* hybridization (smFISH). Arrowheads in the overlay image indicate AP neurons that expresses all the three genes: *Glp1r*, *Gfral* and *Il6ra*. **b.** Confocal images showing the expression of different genes in AP and NTS cells, detected with smFISH. *Fos* was induced after IL-6 retro-orbital injection. AP, area postrema; NTS, nucleus tractus solitarii.

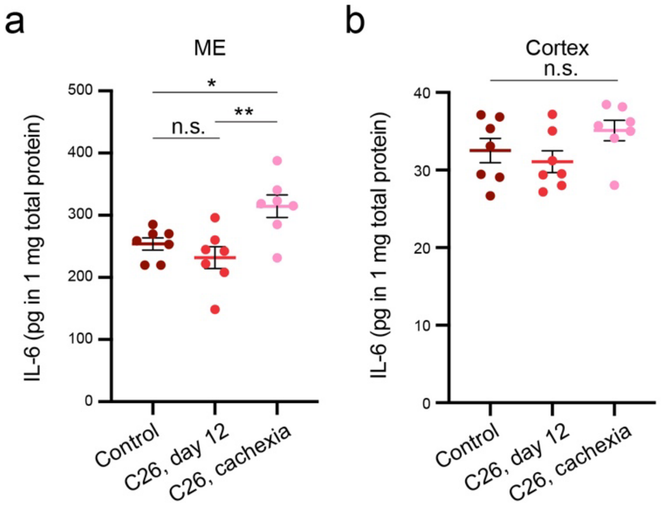

Supplementary Figure 3 | IL-6 levels in different brain areas during C26 cancer progression. IL-6 levels were normalized to total protein levels. **a.** IL-6 levels in the median eminence (ME) during cancer progression (n = 7 mice in each group; control vs. C26 cachexia, F = 3.892, *P = 0.0334; C26 day 12 vs. C26 cachexia, F = 5.305, **P = 0.0040; control vs. C26 day 12, nonsignificant (n.s., P > 0.05); one-way ANOVA followed by Tukey's post-hoc test). **b**. IL-6 levels in the cortex during cancer progression (n = 7 mice in each group, n.s. (P > 0.05), one-way ANOVA).

Data are presented as mean ± s.e.m. Source data are provided as a Source Data file.

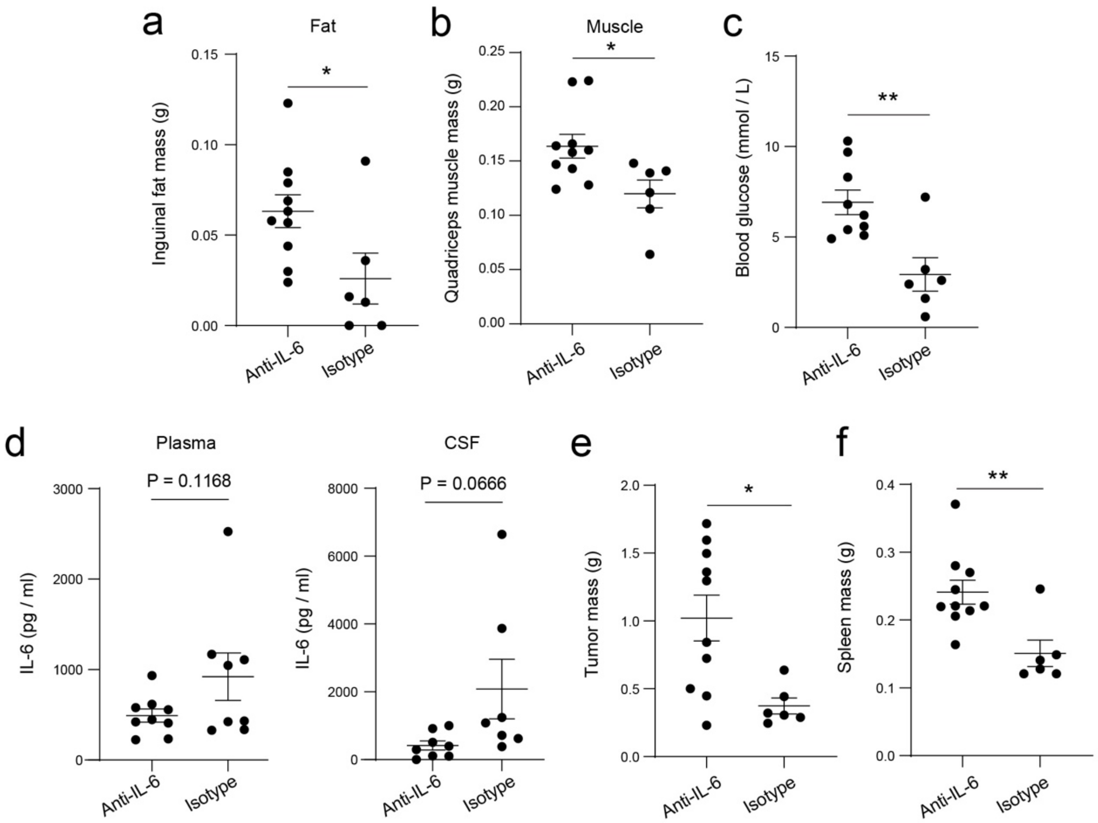

Supplementary Figure 4 | Intracerebroventricular (i.c.v.) infusion of anti-IL-6 antibody improves the physiological conditions of mice despite tumor growth in the C26 cancer model. a. Inguinal fat mass (anti-IL-6 group, n = 10, isotype control group, n = 6; t = 2.334, *P = 0.035, t test). **b.** Quadriceps muscle mass (anti-IL-6 group, n = 10, isotype control group, n = 6; t = 2.538, *P = 0.0237, t test). **c.** Blood glucose levels at the endpoint (anti-IL-6 group, n = 9 mice, isotype control group, n = 6 mice; t = 3.555, **P = 0.0035, t test). **d.** IL-6 levels in the plasma (left) and cerebrospinal fluid (CSF; right). Plasma IL-6: anti-IL-6 group, n = 9 mice, isotype control group, n = 8 mice; t = 1.664, P = 0.1168; CSF IL-6: anti-IL-6 group, n = 8 mice, isotype control group, n = 7 mice; t = 2.002, P = 0.0666; t test. **e.** Tumor mass (anti-IL-6 group, n = 10, isotype control group, n = 6; t = 2.864, *P = 0.0125, t test). **f.** Spleen mass (anti-IL-6 group, n = 10, isotype control group, n = 6; t = 3.274, **P = 0.0055, t test).

Data are presented as mean ± s.e.m. Source data are provided as a Source Data file.

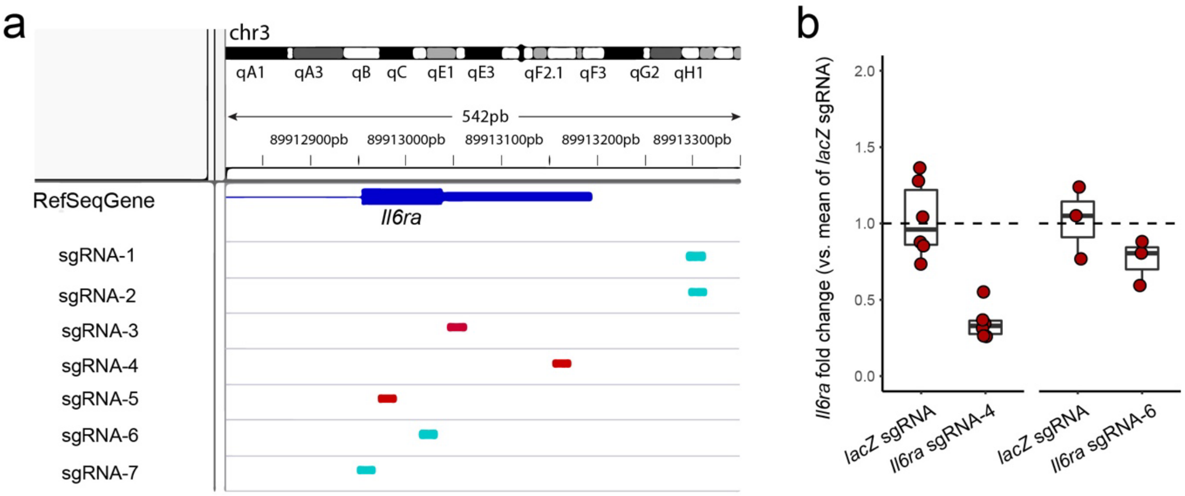

Supplementary Figure 5 | Design and characterization of sgRNAs for the CRISPR/dCas9 system to suppress *Il6ra* expression. a. Transcription start site (TSS) targeting positions of the different sgRNAs. Cyan, template strand; red, non-template strand. **b.** *In vitro* characterization of the efficacy of the CRISPR/dCas9 system with different sgRNAs. Plasmids expressing dCas9-KRAB-MeCP2 and each of the sgRNAs were co-transfected into mHypoA cell line. 60 hours after the transfection, the expression of *Il6ra* in these cells was measured by qPCR (*Il6ra* sgRNA-4, n = 6 plates, *lacZ* sgRNA, n = 6 plates, P = 0.0005785; *Il6ra* sgRNA-6, n = 3 plates, *lacZ* sgRNA, n = 3 plates, P = 0.1965; t test).

Data in b are presented as mean ± s.e.m. Source data are provided as a Source Data file.

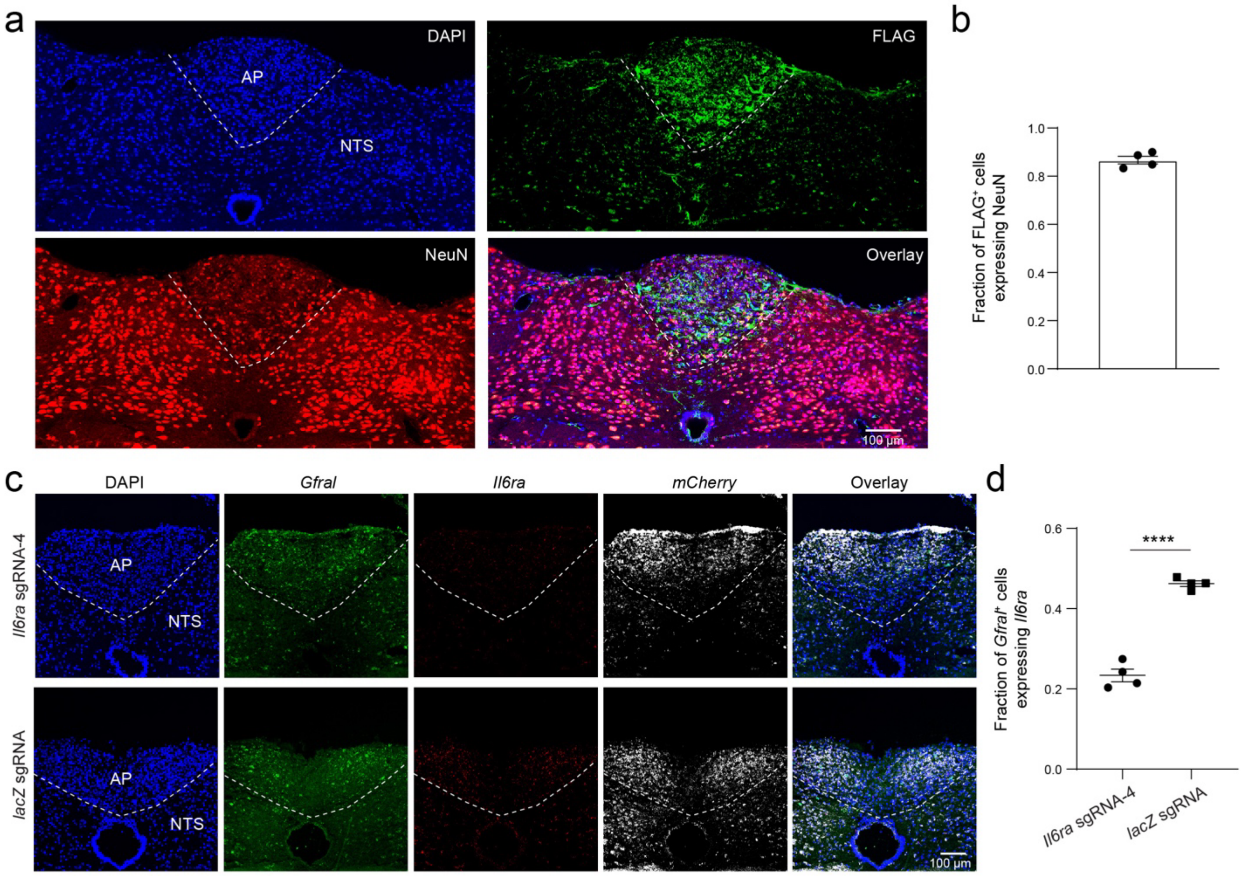

Supplementary Figure 6 |In vivo validation of *Il6ra* suppression in AP neurons by the CRISPR/dCas9 interference system. a. Confocal immunohistochemical images showing the colocalization between NeuN and FLAG in the AP. FLAG indicates the expression of dCas9-KRAB-MeCP2. **b.** Quantification of the colocalization between NeuN and FLAG in the AP (n = 4 mice). **c.** Confocal images showing the expression of *Il6ra*, *Gfral* and *mCherry* (which indicates the expression of sgRNA) in AP cells, detected with smFISH in *Il6ra* sgRNA-4 group (top) and *lacZ* sgRNA (control) group (bottom). **d.** Quantification of the fractions of *Gfral*$^+$ neurons expressing *Il6ra* in the AP of individual mice (*lacZ* sgRNA group, n = 4, *Il6ra* sgRNA-4 group, n = 4; t = 13.22, ****P = 0.000012, t test). AP, area postrema; NTS, nucleus tractus solitarii.

Data in b, d are presented as mean ± s.e.m. Source data are provided as a Source Data file.

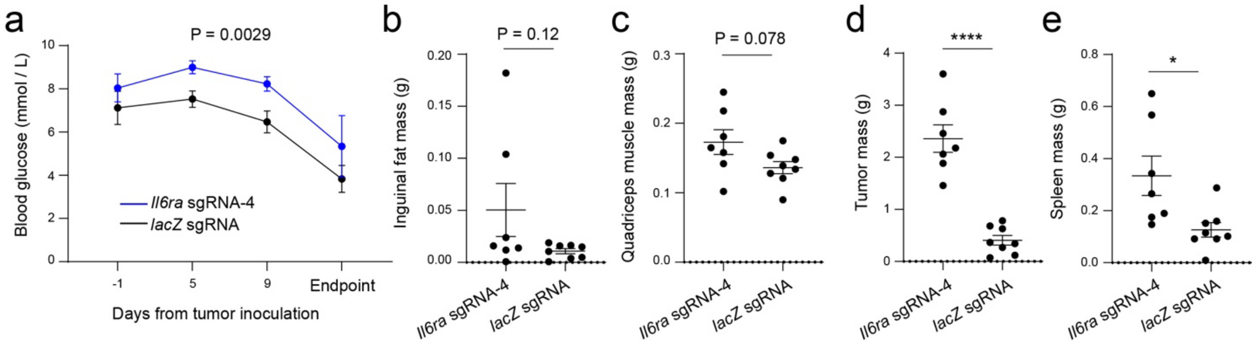

Supplementary Figure 7 | Suppression of *Il6ra* expression in AP neurons improves the physiological conditions of mice despite tumor growth in the C26 cancer model. *Il6ra* sgRNA-4 group, n = 7 mice, *lacZ* sgRNA group, n = 8 mice. **a.** Blood glucose levels at different time points (F(1,49) = 9.834, P = 0.0029; two-way repeated-measures ANOVA with Sidak's post hoc test). **b.** Inguinal fat mass (t = 1.657, P = 0.12, t test). **c.** Quadriceps muscle mass (t = 1.92, P = 0.078, t test). **d.** Tumor mass (t = 7.32, ****P = 5.86 × 10^{-6}, t test). **e.** Spleen mass (t = 2.71, P = 0.018, t test).

Data are presented as mean ± s.e.m. Source data are provided as a Source Data file.

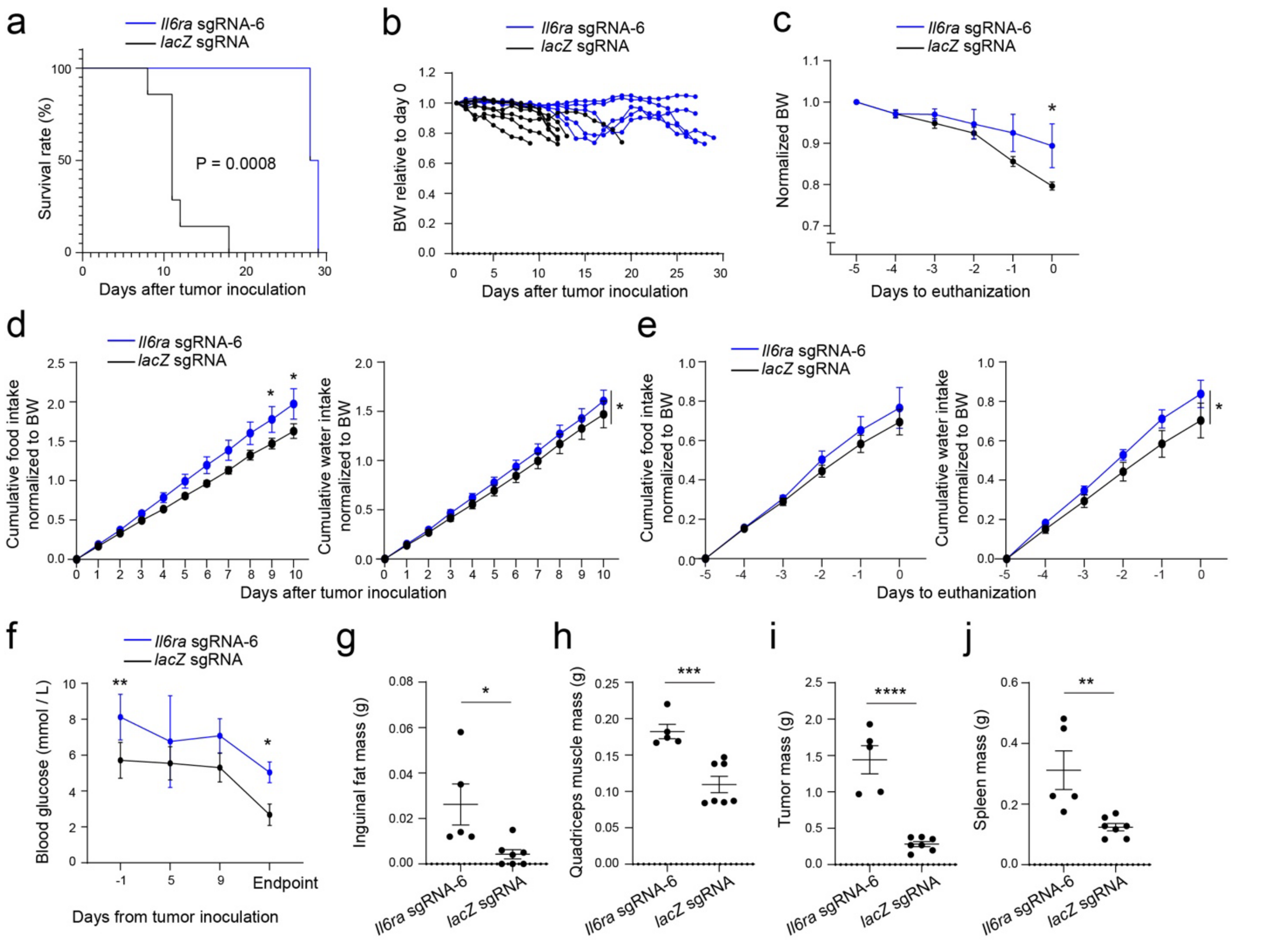

Supplementary Figure 8 | Suppression of *Il6ra* expression in AP neurons attenuates cachexia in the C26 cancer model. *Il6ra* sgRNA-6 group, n = 5 mice, *lacZ* sgRNA group, n = 7 mice. **a.** Survival curves of the mice after tumor inoculation (P = 0.0008, Mantel-Cox test). **b.** Bodyweight of individual mice relative to their bodyweight on the day of tumor inoculation. **c.** Average of bodyweight normalized to that on day -5 ($F(1,60) = 8.287$, $P = 0.0055$; day 0, *P = 0.011, two-way repeated-measures (RM) ANOVA with Sidak's post hoc test). **d.** Normalized cumulative food (left) and water (right) intake of the mice after tumor inoculation (food, $F(1,110) = 30.28$, $P = 2.47 \times 10^{-7}$, *P = 0.043, *P = 0.013; water, $F(1,110) = 5.146$, $P = 0.0253$; two-way RM ANOVA with Sidak's post hoc test). **e.** Normalized cumulative food (left) and water (right) intake of the mice before being euthanized (food, $F(1,60) = 2.051$, $P = 0.158$; water, $F(1,60) = 6.396$, $P = 0.0141$; two-way RM ANOVA with Sidak's post hoc test). **f.** Blood glucose levels at different time points ($F(1,38) = 29.7$, $P = 3.24 \times 10^{-6}$, *P = 0.013, **P = 0.0056, two-way RM ANOVA with Sidak's post hoc test). **g.** Inguinal fat mass (t = 2.78, *P = 0.0195, t test).

h. Quadriceps muscle mass (t = 4.65, ***P = 0.0009, t test). **i.** Tumor mass (t = 7, ****P = 0.000037, t test). **j.** Spleen mass (t = 3.43, **P = 0.0064, t test).

Data in c-j are presented as mean ± s.e.m. Source data are provided as a Source Data file.

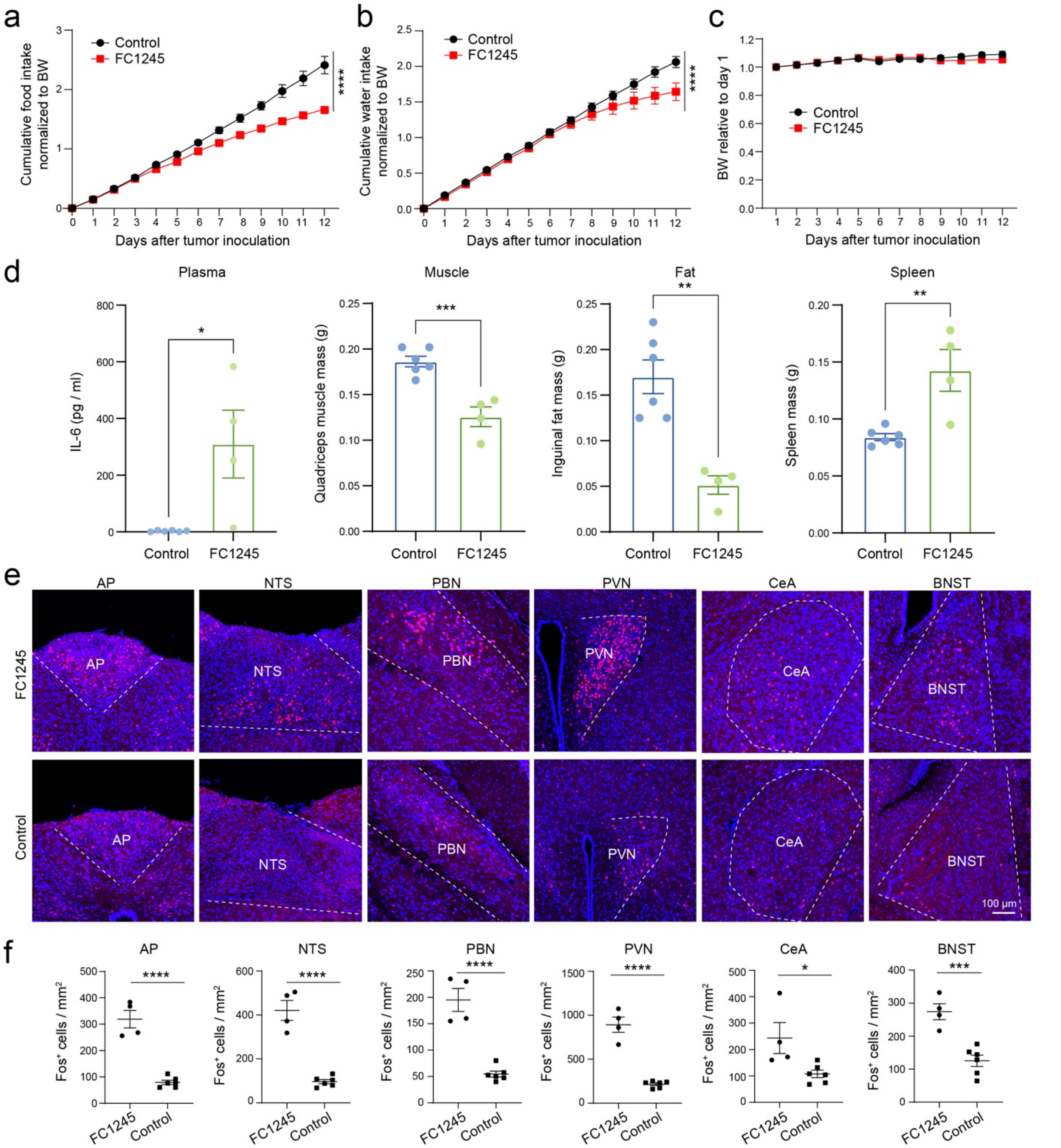

Supplementary Figure 9 |Characterization of cachectic phenotypes in the pancreatic cancer model. a-c. Normalized food intake (a), water intake (b) and bodyweight (c) in mice bearing the FC1245 tumor and control mice. FC1245 tumor group, n = 4 mice, sham control group, n = 6 mice. Bodyweight, $F(1,96) = 1.098$, $P = 0.3$; food intake, $F(1,104) = 95.33$, $P < 10^{-15}$; water intake, $F(1,104) = 22.43$, $P = 6.92\times10^{-6}$; two-way repeated-measures ANOVA with Sidak's post hoc test. **d.** The effects of the tumor on plasma IL-6 levels, muscle weight, fat weight and spleen

weight (FC1245 tumor group, n = 4 mice, sham control group, n = 6 mice; IL-6, t = 3.25, *P = 0.0117; muscle, t = 5.41, ***P = 0.0006; fat, t = 4.87, **P = 0.0012; spleen, t = 3.91, **P = 0.0045; unpaired t test). **e.** Confocal immunohistochemical images showing Fos expression in different brain areas in tumor bearing mice (top) and control mice (bottom). **f.** Quantification of Fos expression in different brain areas (FC1245 tumor group, n = 4 mice, sham control group, n = 6 mice; AP, t = 8.52, ****P = 0.00003; NTS, t = 8.64, ****P = 2.5×10^{-5}; PBN, t = 7.58, ****P = 6.44×10^{-5}; PVN, t = 9.47, ****P = 0.00001; CeA, t = 2.73, *P = 0.0257; BNST, t = 5.2, ***P = 0.0008; unpaired t test). AP, area postrema; NTS, nucleus tractus solitarii; PBN, parabrachial nucleus; PVN, paraventricular nucleus of hypothalamus; CeA, central amygdala; BNST, bed nucleus of the stria terminalis.

Data in a-d & f are presented as mean ± s.e.m. Source data are provided as a Source Data file.

Lentiviral preparation	Physical titer (genomic copies/uL)	Functional titer (transducing units/uL)
FLAG-dCas9-KRAB-MeCP2 (prep 1)	3.0E+11	1.2E+07
FLAG-dCas9-KRAB-MeCP2 (prep 2)	8.0E+10	1.1E+07
Il6ra sgRNA-4	3.0E+12	1.4E+08
Il6ra sgRNA-6	5.0E+11	3.8E+07
lacZ sgRNA	3.0E+12	8.8E+07

Supplementary Table 1. Lentiviral production for CRISPR/dCas9 interference. Titers of the Lentiviral preps used for the experiments in this study are provided.
