# Supplementary material for: Area postrema neurons mediate interleukin-6 function in cancer cachexia
Source: Nat Commun. 2024 Jun 1;15:4682. doi: 10.1038/s41467-024-48971-1 (PMC11144211; doi:10.1038/s41467-024-48971-1)
Supplement: Supplementary file 2 — Reporting Summary [file 41467_2024_48971_MOESM2_ESM.pdf]

nature portfolio

Corresponding author(s): Bo Li

Last updated by author(s): Apr 26, 2024

Reporting Summary

Nature Portfolio wishes to improve the reproducibility of the work that we publish. This form provides structure for consistency and transparency in reporting. For further information on Nature Portfolio policies, see our Editorial Policies and the Editorial Policy Checklist.

Statistics

For all statistical analyses, confirm that the following items are present in the figure legend, table legend, main text, or Methods section.

n/a	Confirmed	
☐	☒	The exact sample size (*n*) for each experimental group/condition, given as a discrete number and unit of measurement
☐	☒	A statement on whether measurements were taken from distinct samples or whether the same sample was measured repeatedly
☐	☒	The statistical test(s) used AND whether they are one- or two-sided<br>*Only common tests should be described solely by name; describe more complex techniques in the Methods section.*
☒	☐	A description of all covariates tested
☐	☒	A description of any assumptions or corrections, such as tests of normality and adjustment for multiple comparisons
☐	☒	A full description of the statistical parameters including central tendency (e.g. means) or other basic estimates (e.g. regression coefficient) AND variation (e.g. standard deviation) or associated estimates of uncertainty (e.g. confidence intervals)
☐	☒	For null hypothesis testing, the test statistic (e.g. *F*, *t*, *r*) with confidence intervals, effect sizes, degrees of freedom and *P* value noted<br>*Give P values as exact values whenever suitable.*
☒	☐	For Bayesian analysis, information on the choice of priors and Markov chain Monte Carlo settings
☒	☐	For hierarchical and complex designs, identification of the appropriate level for tests and full reporting of outcomes
☒	☐	Estimates of effect sizes (e.g. Cohen's *d*, Pearson's *r*), indicating how they were calculated

Our web collection on statistics for biologists contains articles on many of the points above.

Software and code

Policy information about availability of computer code

Data collection	Confocal images were collected via Zen 2011 software of Zeiss. sgRNAs targeting the Il6ra transcription start site (TSS) were designed using CHOPCHOP(Labun, K. et al. Nucleic Acids Res 47,2019). Whole-cell patch clamp recording from area postrema neurons was obtained with Multiclamp 700B amplifiers and pCLAMP 10 software (version 10.2.0.12, Molecular Devices, Sunnyvale, California, USA)
Data analysis	Confocal data for histology were analyzed using ImageJ (VI.52k, NIH). Statistical analyses were performed and plotted with Matlab(R2015b, The MathWorks, Inc., Natick, Massachusetts, USA) or Prism7 (Graph Pad).

For manuscripts utilizing custom algorithms or software that are central to the research but not yet described in published literature, software must be made available to editors and reviewers. We strongly encourage code deposition in a community repository (e.g. GitHub). See the Nature Portfolio guidelines for submitting code & software for further information.

Data

Policy information about availability of data

All manuscripts must include a data availability statement. This statement should provide the following information, where applicable:

- Accession codes, unique identifiers, or web links for publicly available datasets
- A description of any restrictions on data availability
- For clinical datasets or third party data, please ensure that the statement adheres to our policy

The authors declare that the data supporting the findings of this study are available within the paper and its supplementary information files. Source data are provided with this paper.

Research involving human participants, their data, or biological material

Policy information about studies with human participants or human data. See also policy information about sex, gender (identity/presentation), and sexual orientation and race, ethnicity and racism.

Reporting on sex and gender	N/A
Reporting on race, ethnicity, or other socially relevant groupings	N/A
Population characteristics	N/A
Recruitment	N/A
Ethics oversight	N/A

Note that full information on the approval of the study protocol must also be provided in the manuscript.

Field-specific reporting

Please select the one below that is the best fit for your research. If you are not sure, read the appropriate sections before making your selection.

☒ Life sciences ☐ Behavioural & social sciences ☐ Ecological, evolutionary & environmental sciences

For a reference copy of the document with all sections, see nature.com/documents/nr-reporting-summary-flat.pdf

Life sciences study design

All studies must disclose on these points even when the disclosure is negative.

Sample size	No statistical methods were used to pre-determine sample sizes but our sample sizes are similar to those reported in previous publications (Carlos A Campos et al., 2017, Nature Neuroscience, Olson, B. et al. 2021, Nature Communications).
Data exclusions	After histological inspection, the mice in which viral injection was mis-targeted were excluded.
Replication	To ensure that results are reproducible, we included detailed methods, sources of reagents and protocols for all the experiments. All behavioral experiments were successfully repeated with the indicated numbers of mice. Individual data points and error bars were presented in the figures. All imaging data were collected from the specified numbers of cells and mice. All histological experiments were successfully repeated. Exact sample sizes are provided in the figure legends and extended data figure legends.
Randomization	All mice were randomly assigned to different groups.
Blinding	The experiment and analysis in Supplementary Fig. 4 were performed blind. Other data collection and analysis were not performed blind to the conditions of the experiments. However, equal parameter and process were applied for all groups. Further, behavioral experiments were performed under consistent conditions and the related data were collected and analyzed in an unbiased way.

Reporting for specific materials, systems and methods

We require information from authors about some types of materials, experimental systems and methods used in many studies. Here, indicate whether each material, system or method listed is relevant to your study. If you are not sure if a list item applies to your research, read the appropriate section before selecting a response.

Materials & experimental systems

n/a	Involved in the study	
☐	☒	Antibodies
☐	☒	Eukaryotic cell lines
☒	☐	Palaeontology and archaeology
☐	☒	Animals and other organisms
☒	☐	Clinical data
☒	☐	Dual use research of concern
☒	☐	Plants

Methods

n/a	Involved in the study	
☒	☐	ChIP-seq
☒	☐	Flow cytometry
☒	☐	MRI-based neuroimaging

Antibodies

Antibodies used	The primary antibodies and dilutions used in this study were: rabbit anti-Fos (1:500, Santa Cruz, sc-52; 1:1000, Cell Signaling Technology, #2250), mouse anti-FLAG (1:1000, Sigma-Aldrich, F1804), rabbit anti-NeuN (1:200; Abcam, ab177487), rabbit anti-mCherry (1:1,000; Abcam, ab167453, GR3213077-3). The fluorophore-conjugated secondary antibodies and dilutions used were Alexa Fluor 488 goat anti-rabbit IgG (H + L; 1:500; A-11008, Invitrogen), Alexa Fluor 647 goat anti-rabbit IgG (H + L; 1:500; A-21244, Invitrogen), Alexa Fluor 594 goat anti-mouse IgG (H + L; 1:500; A-11005, Invitrogen).
Validation	All the primary and secondary antibodies were validated by extensive previous studies (e.g., Stephenson-Jones et al, 2016, Nature; Zhang and Li, 2018, Nature Communications; Stephenson-Jones et al, 2020, Neuron), and by the manufacturers.

Eukaryotic cell lines

Policy information about cell lines and Sex and Gender in Research

Cell line source(s)	Lewis lung carcinoma cell line (CRL-1642) obtained from American Type Culture Collection (ATCC). C26 cell line originally obtained from Ohio State University Medical Center. The FC1245 clonogenic cell line used in the pancreatic ductal adenocarcinoma (PDAC) model was generously provided by David Tuveson at Cold Spring Harbor Laboratory.
Authentication	C26 cell line has been authenticated by long-reads sequencing. FC1245 and LLC has not been authenticated in our hands. However, they have been used by many labs for studying cancer cachexia [e.g., Michaelis, K. A. et al. Establishment and characterization of a novel murine model of pancreatic cancer cachexia. 8, 824-838, doi:https://doi.org/10.1002/jcsm.12225 (2017). Olson, B. et al. Lipocalin 2 mediates appetite suppression during pancreatic cancer cachexia. Nature Communications 12, 2057, doi:10.1038/s41467-021-22361-3 (2021). Campos, C. A. et al. Cancer-induced anorexia and malaise are mediated by CGRP neurons in the parabrachial nucleus. Nat Neurosci 20, 934-942, doi:10.1038/nn.4574 (2017)].
Mycoplasma contamination	C26 , FC1245 and LLC cell lines tested negative for Mycoplasma contamination.
Commonly misidentified lines (See ICLAC register)	No commonly misidentified lines were used.

Animals and other research organisms

Policy information about studies involving animals; ARRIVE guidelines recommended for reporting animal research, and Sex and Gender in Research

Laboratory animals	Male mice aged 2–4 months were used in all the experiments. Before the experiments, mice were housed under a 12-h light/dark cycle (7 a.m. to 7 p.m. light) in groups of 2–5 animals, with a room temperature (RT) of 22 °C and humidity of 50%. Food and water were available ad libitum. Regular chow (PicoLab rodent diet 20, 5053*; physiological value, 3.43 kcal g–1) were provided to all the animals during feeding experiments. When measuring food and water intake and after the surgery (i.e., canulation or tumor inoculation), mice were singly housed in the metabolic cages. All experimental procedures were approved by the Institutional Animal Care and Use Committee of Cold Spring Harbor Laboratory and performed in accordance with the US National Institutes of Health guidelines. The Balbc mice (strain number: 000651) and C57BL/6J (strain number: 000664) were purchased from Jackson laboratory. The Gfral-p2a-Cre mice was generated by Stephen Liberles.
Wild animals	The study did not involve wild animals.
Reporting on sex	Only male animals were used in this study.
Field-collected samples	The study did not involve samples collected from the field.
Ethics oversight	Institutional Animal Care and Use Committee of Cold Spring Harbor Laboratory.

Note that full information on the approval of the study protocol must also be provided in the manuscript.

Plants

Seed stocks	N/A
Novel plant genotypes	N/A
Authentication	N/A
